# Influence of Food Characteristics and Food Additives on the Antimicrobial Effect of Garlic and Oregano Essential Oils

**DOI:** 10.3390/foods6060044

**Published:** 2017-06-10

**Authors:** Juan García-Díez, Joana Alheiro, Ana Luísa Pinto, Luciana Soares, Virgílio Falco, Maria João Fraqueza, Luís Patarata

**Affiliations:** 1 CECAV, Centro de Ciência Animal e Veterinária, Universidade de Trás-os-Montes e Alto Douro, Quinta de Prados, Vila Real 5000-801, Portugal; jo_alheiro@hotmail.com (J.A.); pinto_aluisa@hotmail.com (A.L.P.); lucimlsoares@hotmail.com (L.S.); lpatarat@utad.pt (L.P.); 2 CQ-VR, Centro de Química-Vila Real (CQ-VR), Universidade de Trás-os-Montes e Alto Douro, Quinta de Prados, Vila Real 5000-801, Portugal; vfalco@utad.pt; 3 CIISA, Faculty of Veterinary Medicine, University of Lisbon, Avenida da Universidade Técnica, Pólo Universitário do Alto da Ajuda, Lisbon 1300-477, Portugal; mjoaofraqueza@fmv.ulisboa.pt

**Keywords:** essential oil, *Salmonella* spp., *Listeria monocytogenes*, food additives, food composition

## Abstract

Utilization of essential oils (EOs) as antimicrobial agents against foodborne disease has gained importance, for their use as natural preservatives. Since potential interactions between EOs and food characteristics may affect their antimicrobial properties, the present work studies the influence of fat, protein, pH, a_w_ and food additives on the antimicrobial effect of oregano and garlic EOs against *Salmonella* spp. and *Listeria monocytogenes.* Results showed that protein, pH, a_w_, presence of beef extract, sodium lactate and nitrates did not influence their antimicrobial effect. In contrast, the presence of pork fat had a negative effect against both EOs associated with their dilution of the lipid content. The addition of food phosphates also exerts a negative effect against EOs probably associated with their emulsification properties as observed with the addition of fat. The results may help the food industry to select more appropriate challenges to guarantee the food safety of foodstuffs.

## 1. Introduction

Use of essential oils (EOs) as flavoring agents by the food industry is common. However, their antimicrobial properties against foodborne pathogens have increased their interest as a source of natural preservatives [1,2]. In dry-cured meat products, utilization of EOs has gained interest in the potential control of pathogens. Considering the strong aromatization of these sausages, resulting from the smoking process and seasonings [3,4], EOs as antimicrobial agents could be used since it is expected that the sensory impact of EOs will be mitigated by the global aroma of the product. 

The antimicrobial effect of EOs is different in foodstuffs than in in vitro studies [5]. Since factors such as pH, a_w_, food composition or potential interactions with food additives influence the antimicrobial effect of the EOs [6], a previous in vitro screening against specific foodborne pathogens tested in specific food-like growth media could be an important approach to highlight potential interactions between EOs and food characteristics. Thus, the present work is aimed at studying the influence of fat and protein levels, different pH and a_w_ and the presence of sodium nitrite, commercial phosphates and sodium lactate on the inhibitory properties of oregano and garlic EOs against *Salmonella* spp. and *L. monocytogenes* isolated from meat products.

## 2. Material and Methods

### 2.1. Gas Chromatography-Mass Spectrometry Analysis of Essential Oils 

EOs of the spices of garlic (*Allium sativum*, bulbs) and oregano (*Origanum vulgare*, leaves), commonly used to manufacture dry-cured meat products, as reported by Melo et al. [7], were selected. All EOs and their technical characteristics were kindly provided by Ventós Chemicals (Barcelona, Spain). The Gas chromatography-mass spectrometry analysis was carried out as described elsewhere [8].

### 2.2. Microorganisms and Growth Conditions

Stock cultures of *Salmonella* spp. and *L. monocytogenes* (Table 1) isolated either from traditional dry-cured fermented sausages during their manufacturing or from the environment of their production were identified by a species-specific PCR technique [9]. Each microorganism was maintained at −18 °C and subcultured twice in brain heart infusion (BHI-Biokar, Beauvais, France). Incubation for *Salmonella* spp. was done at 37 °C, while *L. monocytogenes* was incubated at 30 °C. Overnight cultures in BHI were streaked in BHI agar and incubated during 18 to 24 h. To prepare the inoculum for the sensitivity test to EOs, a suspension of isolated colonies in BHI agar was made in NaCl 0.85%. The turbidity of the suspension was adjusted to 0.5 McFarland standard (Biomerieux, Marcy-l’Etoile, France).

### 2.3. Antimicrobial Effect on Disk Diffusion Assay

The antimicrobial effect of EOs was screened by the disk diffusion assay (DDA) as described by Zaika [10], but with some modifications. Petri plates prepared with 20 mL of Mueller–Hinton agar (MHA, Biokar. Beauvais, France) were dried, and 100µL of standardized inoculum suspension (ca.8 log CFU/mL) were poured and uniformly spread. Filter paper disks (Whatman No.1, 6-mm diameter, GE Healthcare, Madison, WI, USA) containing 20 μL of each EOs were applied to the surface of the previously seeded agar plates of MHA. The plates were kept at 4 °C for 2 h to allow dispersion and were incubated overnight at the optimum growth temperature of each microorganism under study (as above mentioned) during 18 to 24 h. The antimicrobial activity was visually evaluated as the inhibition zone surrounding the disk, and their diameters, including the disk diameters, were measured in mm. The results representing the net zone of inhibition including the diameter (6 mm) of the paper disk are the mean of 3 determinations for each isolate tested.

### 2.4. Determination of MIC and MBC

The minimum inhibitory concentration (MIC) and minimum bactericidal concentration (MBC) were studied for both EOs. The dilutions of the EOs were established based on the inhibitory profile with the DDA. The assay was based on the procedure of the Clinical and Laboratory Standards Institute [11] with 96-well microtiter plates. The MIC was considered the lowest concentration of EOs at which bacteria failed to grow, as detected by the unaided eye, matching with the negative control without inoculation included in the test. The visual evaluation was complemented with the seeding of a 10-µL loop in MHA to confirm the absence of growth. To evaluate the MBC, 10 µL of each well, in which no microbial growth was observed, were spread into MHA plates and incubated for 24 h. The MBC was considered as the lowest concentration determining a reduction in the population of 99.9%.

### 2.5. Preparation of Food Model Media

#### Influence of Fat, Protein, pH and a_w_

The effect of different levels of fat and protein, pH and a_w_ on the antimicrobial effect of EOs was performed using a food model media. *L. monocytogenes* or *Salmonella* spp. were used as indicator strains. EOs of oregano and garlic were used at 0.05% and 0.005%, respectively. The influence of 2.5%, 5% and 10% of fat was studied by the addition of pork fat, purchased at a local supermarket and previously sterilized by autoclaving before being added to Mueller-Hinton broth supplemented with 1% agar (MHB, Biokar. Beauvais, France). The fat was emulsified with the broth with an Ultra-Turrax (IKA, Staufen, Germany). The influence of protein was tested by the addition of beef extract (Oxoid, Hampshire, UK) at 10%, 15% and 20% to MHB. The influence of pH was tested by the addition of lactic acid (Panreac Applichem, Barcelona, Spain) to MHB to achieve a final pH of 4.5, 5.5 or 6.5 (Crison, Barcelona, Spain) with a penetration probe (Mettler-Toledo, Giesen, Germany). The influence of a_w_ was tested by adjusting the MHB to a final a_w_ of 0.91, 0.94, 0.97 by the addition of NaCl as described by Troller et al. [12]. The a_w_ was measured in a Hygroscope DT apparatus (Rotronic, Zurich, Switzerland) with a WA40 cell maintained at 20±2 °C. All of the levels of fat and protein concentration, pH and a_w_ were selected to simulate the physical and chemical conditions of a traditional dry-cured meat product.

### 2.6. Influence of Food Additives (Sodium Nitrite, Commercial Phosphates and Sodium Lactate)

The influence of food additives on the antimicrobial effect of EOs of oregano and garlic was assessed by the addition of nitrites, phosphates and sodium lactate to MHB as follows: 150 ppm sodium nitrite (Merck, Darmstadt, Germany), 0.5% commercial phosphate (E451 plus E452, BK Giulini, Mannhein, Germany) and 3.3% sodium lactate (Sigma-Aldrich, St. Luis, MO, USA).

### 2.7. Microbial Preparation

Two strains of *Salmonella* spp. and two of *L. monocytogenes*, identified by a species-specific PCR technique as indicated above [9], were used in the experiment. The strains used (Table 1) did not present sensibility differences against the antimicrobial effect of EOs of oregano and garlic as reported elsewhere [8]. Single strain cultures of each pathogen were inoculated, in duplicate, in test tubes with 10 mL of culture medium prepared with the specific modification. The inoculation was made to achieve an initial contamination of about 5.7 log CFU/mL. Inoculated tubes were incubated at 37 °C for *Salmonella* spp. or 30 °C for *L. monocytogenes.* Counts were performed after 4, 8, 12, 24 and 36 h of incubation by serial ten-fold dilution prepared from 1 mL of the culture in xylose lysine desoxycholateagar (Oxoid, Hampshire, UK) for *Salmonella* spp. and Oxford agar (Oxoid, Hampshire, UK) for *L. monocytogenes*. Tests with food additives were performed only until 24 h of incubation. The experiment was carried out in triplicate, and results are expressed as the log CFU/mL of culture medium.

### 2.8. Statistical Analysis 

The influence of fat and protein levels, a_w_, pH and food additives on the antimicrobial effect of oregano and garlic EOs against each pathogen was carried out by analysis of variance (ANOVA), evaluating the combined effect of the presence of EOs and the level of the composition modification studied, for each incubation time. The Tukey–Kramer test was used to determine the significant differences (*p* < 0.05) among means. Statistical analysis was done with SPSS 19.0 software (SPSS Inc., Chicago, IL, USA) for Windows 8 (Redmont, Washington, DC, USA), considering *p* < 0.05 as statistically significant.

## 3. Results and Discussion

### 3.1. Chemical Composition and Antimicrobial Properties of EOs

Information about the utilization of EOs to improve both the food safety and shelf-life of foodstuffs is scarce and mainly aimed at fresh foodstuffs [13,14,15], although some reports studied its application in meat products [16,17]. Since meat products presented specific characteristics as variable protein and fat levels, pH, a_w_ or additives, the influence of these characteristics on the inhibitory effect of EOs should be previously assessed in vitro to address their further potential application in meat products. Although some works studied the influence of food composition on the antimicrobial effect of EOs in specific foodstuffs [5], the present work is, to the best knowledge of the authors, the first report that studied the influence of food composition and food additives commonly present in meat products.

EOs of garlic and oregano displayed a noticeable inhibitory activity against *Salmonella* spp. and *L. monocytogenes* [18,19] based on their main chemical compounds, thymol and sulfur compounds, respectively [1] (Table 2 and Table 3). Regarding the MIC and the MBC (Table 3), results were in accordance with DDA. The large inhibition halos observed for *L. monocytogenes* for EOs of garlic and oregano were in accordance with the low MIC and MBC values. In contrast, the higher MIC and MBC of garlic essential oil for *Salmonella* spp. were in accordance with the lower halo size.

### 3.2. Effect of Fat, Protein, a_w_, pH and Food Additives (Sodium Nitrite, Sodium Lactate and Food Phosphates)

**Figure 1:**
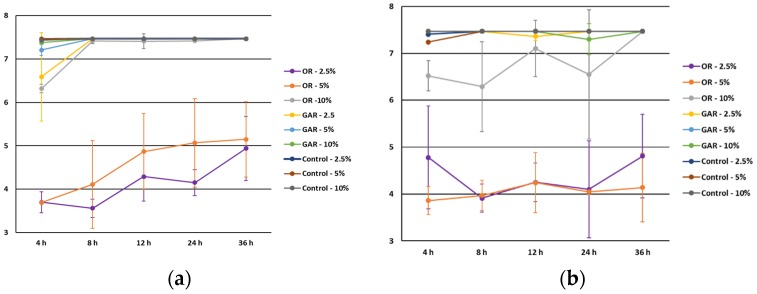
Influence of fat level on the antimicrobial effect of oregano (OR) and garlic (GAR) essential oils against *Salmonella* spp. (**a**) and *Listeria monocytogenes* (**b**).

Regarding the influence of the fat level (Figure 1), the inhibitory effect of EOs of garlic and oregano decreased as the fat level increases. Oregano EOs displayed a noticeable inhibitory effect at a 2.5% and 5% fat level after 4 and 8 h. of incubation. However, 12 h later, counts of *Salmonella* spp. and *L. monocytogenes* were similar in samples with garlic EOs and control. Oregano EOs showed higher antimicrobial activity than garlic EOs (*p* < 0.05), although no statistical differences were observed between samples with garlic EOs and control (*p* > 0.05). A high level of fat exerted a negative effect of the antimicrobial effect of oregano EOs in accordance as reported in the literature [6]. In addition, the antimicrobial effect of oregano essential oil, in the presence of fat, was similar for *Salmonella* spp. and *L. monocytogenes*, contrary to what was reported by Solórzano-Santos and Miranda-Novales [20]. The decrease in the inhibitory activity at high fat levels could be explained by its protective effect or by the dilution effect of EOs in the fat, decreasing the contact between EOs and pathogens [21]. Our results are in accordance with Smith-Palmer et al. [19], who reported a higher inhibition of *L. monocytogenes* and *S. enteritidis* in low-fat soft cheese than in high-fat soft cheese. In addition, the increase of the microbial counts along the cheese ripening is also observed in the current study. The negative effect of fat was also reported by Cava et al. [22], who observed a decrease in the antimicrobial activity of cinnamon and clove EOs against *L. monocytogenes* in whole milk compared to skimmed milk. Moreover, Singh et al. [23] observed similar results against *L. monocytogenes* in low-fat hotdog. The high inhibitory effect observed at the lowest fat level could be achievable by the high contact between EOs and foodborne pathogens after 4 h. However, the decrease in the antimicrobial effect along the study suggests a dispersion of the EOs on the lipid content of the food model media. It suggests that the decrease of the inhibitory effect is caused by a dilution effect. In the case of the EOs of garlic, the reduction of its antimicrobial properties could be associated with the degradation of sulfur compounds by the chemical reactions of lipid oxidation, as suggested by Druum et al. [24].

**Figure 2:**
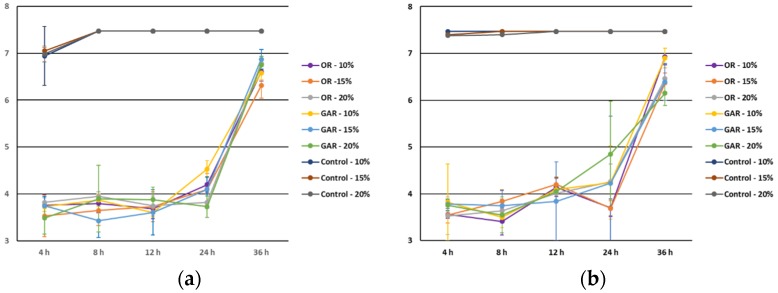
Influence of protein level (Prot) on the antimicrobial effect of oregano (OR) and garlic (GAR) essential oils against *Salmonella* spp. (**a**) and *Listeria monocytogenes* (**b**).

Protein level (Figure 2) did not influence (*p* > 0.05) the antimicrobial effect of oregano and garlic EOs although lower microbial counts were observed at the higher level tested. Other studies showed a reduction of the antimicrobial effect of thyme and oregano EOs in the presence of beef extract [6], as well as in the presence of minced fish [25] against *L. monocytogenes*. This suggests that the presence of high levels of protein could decrease the interaction between EOs and microorganisms due to the formation of a three-dimensional matrix of proteins that acts as a barrier [26,27] or by their hydrophobic properties [28,29]. Thus, the difficult distribution of the EOs in the food model media may explain the scarce antimicrobial effect.

The antimicrobial effect of EOs against *Salmonella* spp. and *L. monocytogenes* was noticeable at the three levels of a_w_ tested and lower than control samples (*p* < 0.001), although the results were similar in samples with EOs (*p* > 0.05).

**Figure 3:**
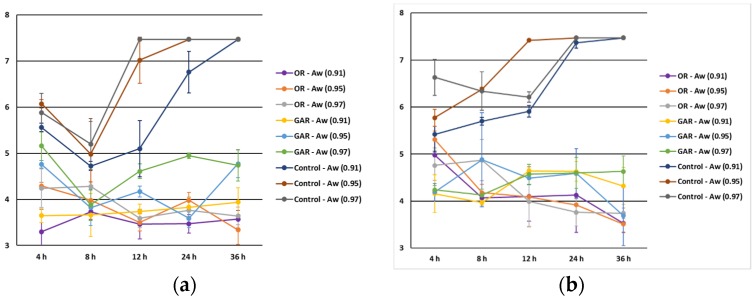
Influence of a_w_ level on the antimicrobial effect of oregano (OR) and garlic (GAR) essential oils against *Salmonella* spp. (**a**) and *Listeria monocytogenes* (**b**).

The influence of a_w_ (Figure 3) on the antimicrobial effect of EOs was slightly higher at reduced a_w_, particularly at 0.91. The a_w_ range for growth of *Salmonella* spp. and *L. monocytogenes* is 0.94 to 0.99 and 0.92 to 0.99, respectively [29,30]. Since *L. monocytogenes* is a Gram-positive bacteria, a higher susceptibility to the antimicrobial effect of Eos was expected due to the absence of the outer membrane that acts as a protective barrier. However, its capacity to adapt to osmotic stress [31] may explain the higher counts compared to *Salmonella* spp. at a_w_ 0.91.

**Figure 4:**
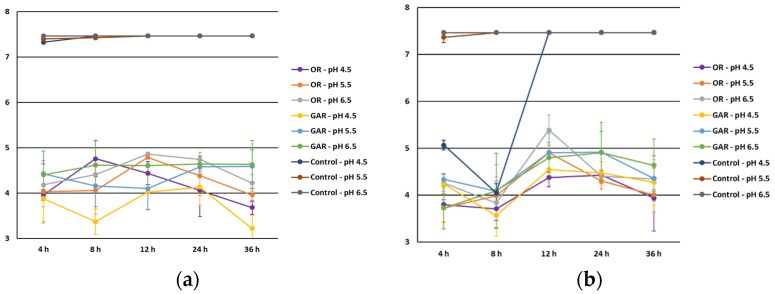
Influence of pH level on the antimicrobial effect of oregano (OR) and garlic (GAR) essential oils against *Salmonella* spp. (**a**) and *Listeria monocytogenes* (**b**).

The influence of pH (Figure 4) on the antimicrobial effect of EOs was similar as observed for a_w_. However, no statistical differences were observed among the three pH levels tested (*p* > 0.05). Lower counts of *Salmonella* spp. and *L. monocytogenes* were observed at pH 4.5 in accordance with Gutierrez et al. [6]. These decreases could be associated with an increase in the hydrophobicity of EOs at low pH that facilitates the dissolution of the lipids presented in the outer membrane of foodborne pathogens [32,33].

**Figure 5:**
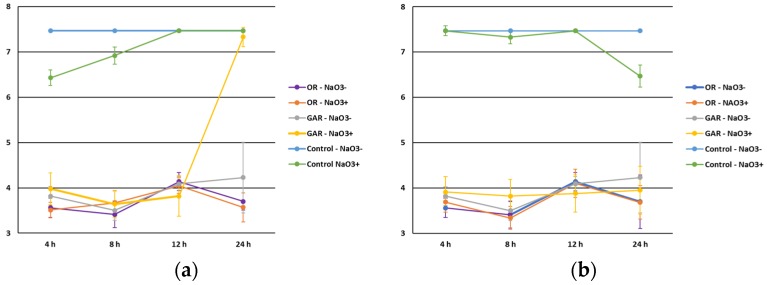
Influence of sodium nitrite (absence, NaNO3^−^; presence, NaNO3^+^) level on the antimicrobial effect of oregano (OR) and garlic (GAR) essential oils against *Salmonella* spp. (**a**) and *Listeria monocytogenes* (**b**).

**Figure 6:**
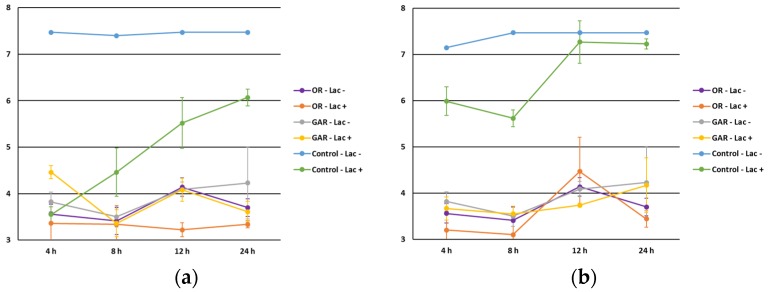
Influence of sodium lactate (absence, Lac−; presence, Lac+) level on the antimicrobial effect of oregano (OR) and garlic (GAR) essential oils against *Salmonella* spp. (**a**) and *Listeria monocytogenes* (**b**).

Regarding food additives, the inhibitory effect of EOs was observed in the presence and absence of sodium nitrite (Figure 5) and sodium lactate (Figure 6). This indicates that a decrease in the microbial counts could not be attributed to a synergic effect with these additives (*p* > 0.05). Sodium nitrite is mainly used as a preservative, to control microbial development, especially *Clostridium botulinum*, although its addition enhances some characteristics of foodstuffs, such as the aroma and typical color of cured meat products, by the formation of nitrosylmyoglobin [34]. Although some works [35] suggested an antimicrobial effect of nitrites against Enterobacteriaceae in dry-cured chorizo, this effect should be carefully interpreted since other factors, such as the decrease in the pH or a_w_ values, with special relevance in this kind of product, may directly affect the survival of foodborne pathogens.

Regarding sodium lactate, lower microbial counts were observed in the presence of EOs. However, the influence of other factors, namely pH (as previously indicated), could be responsible for the antimicrobial effect and not achievable with respect to the additive.

**Figure 7:**
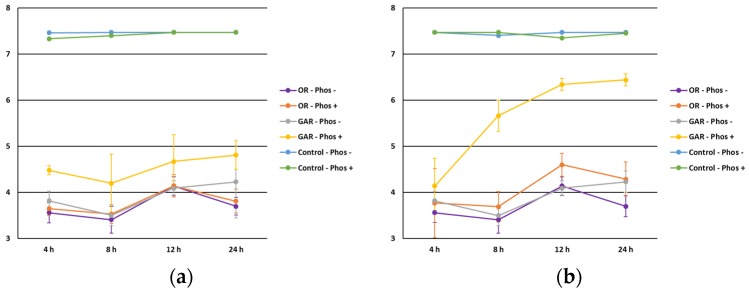
Influence of food phosphates’ (absence, Phos−; presence, Phos+) level on the antimicrobial effect of oregano (OR) and garlic (GAR) essential oils against *Salmonella* spp. (**a**) and *Listeria monocytogenes* (**b**).

Regarding commercial phosphates (Figure 7), the antimicrobial effect against *Salmonella* spp., *L. monocytogenes* and *Escherichia coli* in combination with heat treatment was reported by Dickson et al. [36]. Contrary to what was observed for sodium nitrite and sodium lactate, microbiological counts increased along the study period indicating a potential interaction between food phosphates and EOs, decreasing their availability to act against the foodborne pathogens tested. In addition, the potential role of food phosphates as fat and protein emulsifiers in the manufacturing of meat products could decrease the contact of EOs and microorganisms [1]. Thus, it might be hypothesized that a possible interaction between phosphates and the Mueller–Hinton composition (beef extract and casein) decreases the antimicrobial effect of the EOs by the phenomena previously described in the protein interaction.

## 4. Conclusions

The present work studied the influence of fat, protein, pH, a_w_ and food additives (sodium nitrite, sodium lactate and food phosphates), individually evaluated, on the antimicrobial effect of oregano and garlic EOs against *Salmonella* spp. and *L. monocytogenes*. Although both EOs presented an important antibacterial effect against the foodborne pathogens tested, the results showed that the presence of fat acts as a barrier to its antimicrobial effect probably due to its dilution on the lipid content of the food model media. The level of protein, pH and a_w_ did not influence the antimicrobial effect of EOs, although lower microbial counts were observed at the lowest protein level, a_w_ and pH, respectively. Furthermore, the addition of sodium nitrite and sodium lactate did not influence the inhibitory effect of EOS.

The use of food phosphates could decrease the antimicrobial effect of EOs due to their emulsification properties. The in vitro study of the influence of food characteristic on the antimicrobial effect of EOs could be interesting to the food industry to address the behavior of EOs against specific foodborne pathogens prior their application in foodstuff. Since there is no protocol for evaluating the effect of food additives and essential oils in vitro, the present methodology could be used as an approach for food operators before the utilization of EOs in foodstuffs.

## Figures and Tables

**Table 1:** Strains used in the experiment.

Microorganisms	Strain	Source ^1^
*Salmonella* spp.	MPI-B-S07	Chouriço batter
	EDS-E-S02	Environment of meat products preparation
*L. monocytogenes*	EDS-B-LM02	Chouriço batter
	MPI-E-LM01	Environment of meat products preparation

^1^ Strains isolated from meat products or the environment of its production are from our laboratory collection.

**Table 2:** Chemical composition of essential oils of oregano and garlic determined by GC-MS.

Garlic essential oil		Oregano essential oil	
Compounds	%	Compounds	%
Diallyl sulfide	8.36	á-Pinene	0.27
Methyl allyl disulfide	2.76	á-Terpinolene	0.27
diallyl disulfide	18.86	*p*-cymene	0.99
Methyl allyltrisulfide	9.04	á-Terpinene	1.29
1,3,5 trithiane	0.75	Linalool	0.21
2-vinil-1,3-dithiane	0.75	Thymol	93.34
diallyltrisulfide	33.82	*Trans*-Caryophyllene	0.72
Hexamethylenesulfoxide	0.24	Germacrene	0.13
Methyl allyl disulfide	2.75		
Diallyltetrasulfide	10.97		

**Table 3:** Zones of growth inhibition (mm; mean ± standard deviation) with the DDA (disk diffusion assay), minimal inhibitory concentration (MIC) and minimal bactericide concentration (MBC) of garlic and oregano essential oils against *Salmonella* spp. and *L. monocytogenes*.

Essential Oil	Assay	*Listeria monocytogenes*	*Salmonella* spp.
garlic	DDA (mm)	10.5 ± 1.6	15.03 ± 2.6
	MIC (%)	2	0.0125
	MBC (%)	4	2
oregano	DDA (mm)	46.5 ± 3.2	36.4 ± 1.3
	MIC (%)	0.005	0.005
	MBC (%)	>0.005	>0.005

## References

[1] Hyldgaard M., Mygind T., Meyer R.L. (2012). Essential oils in food preservation: Mode of action, synergies, and interactions with food matrix components. Front. Microbiol..

[2] Tiwari B.K., Valdramidis V.P., O’Donnell C.P., Muthukumarappan K., Bourke P., Cullen P.J. (2009). Application of natural antimicrobials for food preservation. J. Agric. Food Chem..

[3] García-Díez J., Patarata L. (2013). Behaviour of *Salmonella* spp., *Listeria monocytogenes*, and *Staphylococcus aureus* in Chouriço de Vinho, a dry fermented sausage made from wine-marinated meat. J. Food Prot..

[4] Linares M.B., Garrido M.D., Martins C., Patarata L. (2013). Efficacies of Garlic and *L. Sakei* in wine-based marinades for controlling *Listeria monocytogenes* and *Salmonella* spp. inchouriço de vinho, a dry sausage made from wine-marinated pork. J. Food Sci..

[5] Perricone M., Arace E., Corbo M.R., Sinigaglia M., Bevilacqua A. (2015). Bioactivity of essential oils: A review on their interaction with food components. Front. Microbiol..

[6] Gutierrez J., Barry-Ryan C., Bourke P. (2009). Antimicrobial activity of plant essential oils using food model media: Efficacy, synergistic potential and interactions with food components. Food Microbiol..

[7] Hierro E., Fernandez M., de la Hoz L., Toldrá F., Hui Y.H., Astiasaran I., Sebranek J., Talon R. (2015). Mediterranean Products. Handbook of Fermented Meat and Poultry.

[8] García-Díez J., Alheiro J., Falco V., Fraqueza M.J., Patarata L. (2016). Chemical characterization and antimicrobial properties of herbs and spices essential oils against pathogens and spoilage bacteria associated to dry-cured meat products. J. Essent. Oil Res..

[9] Talon R., Lebert I., Lebert A., Leroy S., Garriga M., Aymerich T., Drosinos E.H., Zanardi E., Ianieri A., Fraqueza M.J. (2007). Traditional dry fermented sausages produced in small-scale processing units in Mediterranean countries and Slovakia. 1. Microbial ecosystems of processing environments. Meat Sci..

[10] Zaika L.L. (1987). Spices and herbs: Their antimicrobial activity and its determination. J. Food Saf..

[11] Clinical and Laboratory Standards Institute (2009). Methods for Dilution Antimicrobial Susceptibility Tests for Bacteria That Grow Aerobically. Approved Standard—Eighth Edition. Clin. Lab. Stand. Inst..

[12] Troller J.A., Stinson J.V. (1981). Moisture requirements for growth and metabolite production by lactic acid bacteria. Appl. Environ. Microbiol..

[13] Fratianni F., De Martino L., Melone A., De Feo V., Coppola R., Nazzaro F. (2010). Preservation of chicken breast meat treated with thyme and balm essential oils. J. Food Sci..

[14] Karabagias I., Badeka A., Kontominas M.G. (2011). Shelf life extension of lamb meat using thyme or oregano essential oils and modified atmosphere packaging. Meat Sci..

[15] Soultos N., Tzikas Z., Christaki E., Papageorgiou K., Steris V. (2009). The effect of dietary oregano essential oil on microbial growth of rabbit carcasses during refrigerated storage. Meat Sci..

[16] Dussault D., Dang Vu K., Lacroix M. (2014). In vitro evaluation of antimicrobial activities of various commercial essential oils, oleoresin and pure compounds against food pathogens and application in ham. Meat Sci..

[17] Viuda-Martos M., Ruiz-Navajas Y., Fernández-López J., Pérez-Álvarez J.A. (2010). Effect of added citrus fibreand spice essential oils on quality characteristics and shelf-life of mortadella. Meat Sci..

[18] Benkeblia N. (2004). Antimicrobial activity of essential oil extracts of various onions (*Allium cepa*) and garlic (*Allium sativum*). LWT Food Sci. Technol..

[19] Smith-Palmer A., Stewart J., Fyfe L. (2001). The potential application of plant essential oils as natural food preservatives in soft cheese. Food Microbiol..

[20] Solórzano-Santos F., Miranda-Novales M.G. (2012). Essential oils from aromatic herbs as antimicrobial agents. Curr. Opin. Biotechnol..

[21] Gil A.O., Delaquis P., Russo P., Holley R.A. (2002). Evaluation of antilisterial action of cilantro oil on vacuum packed ham. Int. J. Food Microbiol..

[22] Cava R., Nowak E., Taboada A., Marin-Iniesta F. (2007). Antimicrobial activity of clove and cinnamon essential oils against *Listeria monocytogenes* in pasteurized milk. J. Food Protect..

[23] Singh A., Singh R.K., Bhunia A.K., Singh N. (2003). Efficacy of plant essential oils as antimicrobial agents against *Listeria monocytogenes* in hotdogs. LWT Food Sci. Technol..

[24] Drumm T.D., Spanier A.M. (1991). Changes in the content of lipid autoxidation and sulfur-containing compounds in cooked beef during storage. J. Agric. Food Chem..

[25] Abdollahzadeh E., Rezaei M., Hosseini H. (2014). Antibacterial activity of plant essential oils and extracts: The role of thyme essential oil, nisin, and their combination to control *Listeria monocytogenes* inoculated in minced fish meat. Food Control.

[26] Baranauskien R., Venskutonis P.R., Dewettinck K., Verhe R. (2006). Properties of oregano (*Origanum vulgare* L.), citronella (*Cymbopogonnardus* G.) and marjoram (*Majorana hortensis* L.) flavors encapsulated into milk protein-based matrices. Food Res. Int..

[27] Skandamis P., Tsigarida E., Nychas G.J. (2000). Ecophysiological attributes of *Salmonella typhimurium* in liquid culture and within a gelatin gel with or without the addition of oregano essential oil. World J. Microbiol. Biotechnol..

[28] Burt S. (2004). Essential oils: Their antibacterial properties and potential applications in foods—A review. Int. J. Food Microbiol..

[29] Mølbak K., Olsen J.E., Wegener H.C., Cliver D.O., Potter M., Riemann H.P. (2011). Salmonella infections. Foodborne Infections and Intoxications.

[30] Pagotto F., Corneau N., Farber J., Cliver D.O., Potter M., Riemann R.P. (2011). *Listeria* *monocytogenes*. Foodborne Infections and Intoxications.

[31] Gandhi M., Chikindas M.L. (2007). Listeria: A foodborne pathogen that knows how to survive. Int. J. Food Microbiol..

[32] Skandamis P.N., Nychas G.J.E. (2000). Development and evaluation of a model predicting the survival of *Escherichia coli* O157: H7 NCTC 12900 in homemade eggplant salad at various temperatures, pHs, and oregano essential oil concentrations. Appl. Environ. Microbiol..

[33] Negi P.S. (2012). Plant extracts for the control of bacterial growth: Efficacy, stability and safety issues for food application. Int. J. Food Microbiol..

[34] Cassens R.G. (1997). Composition and safety of cured meats in the USA. Food Chem..

[35] Gonzalez B., Díez V. (2002). The effect of nitrite and starter culture on microbiological quality of “chorizo”—A Spanish dry cured sausage. Meat Sci..

[36] Dickson J.S., Cutter C.G., Siragusa G.R. (1994). Antimicrobial effects of trisodium phosphate against bacteria attached to beef tissue. J. Food Prot..

